# Carbon fixation of CO_2_ via cyclic reactions with borane in gaseous atmosphere leading to formic acid (and metaboric acid); A potential energy surface (PES) study

**DOI:** 10.3389/fchem.2022.1003086

**Published:** 2022-10-05

**Authors:** Marziyeh Mohammadi, Seyyed Amir Siadati, Sheida Ahmadi, Sepideh Habibzadeh, Mohammad Reza Poor Heravi, Zinatossadat Hossaini, Esmail Vessally

**Affiliations:** ^1^ Department of Chemistry, Faculty of Science, Vali-e-Asr University of Rafsanjan, Rafsanjan, Iran; ^2^ Department of Chemistry, Qaemshahr Branch, Islamic Azad University, Qaemshahr, Iran; ^3^ Department of Chemistry, Payame Noor University, Tehran, Iran

**Keywords:** carbon dioxide, atmospheric pollution, borane cycle, reaction channels, CO_2_ fixation, metaboric acid

## Abstract

Carbon dioxide (CO_2_), a stable gaseous species, occupies the troposphere layer of the atmosphere. Following it, the environment gets warmer, and the ecosystem changes as a consequence of disrupting the natural order of our life. Due to this, in the present reasearch, the possibility of carbon fixation of CO_2_ by using borane was investigated. To conduct this, each of the probable reaction channels between borane and CO_2_ was investigated to find the fate of this species. The results indicate that among all the channels, the least energetic path for the reaction is reactant complex (RC) to TS (A-1) to Int (A-1) to TS (A-D) to formic acid (and further meta boric acid production from the transformation of boric acid). It shows that use of gaseous borane might lead to controlling these dangerous greenhouse gases which are threatening the present form of life on Earth, our beautiful, fragile home.

## Graphical Abstract

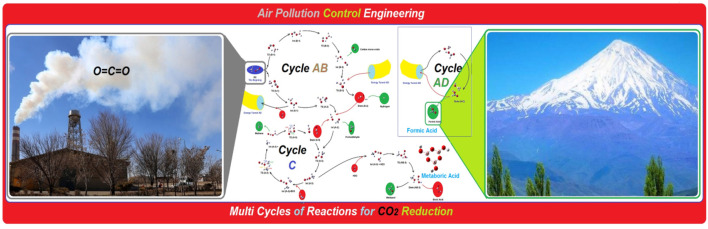

## Introduction

Carbon dioxide has been one of the most dangerous threats to the present order of our lives. As one of the major greenhouse gases, it makes our environment warmer and directly changes the ecosystem of Earth, the only known planet in the cosmos that supports complex life forms (Herndon and Whiteside, 2021).

On the one hand, in spite of the global attempts of scientists, the amount of CO_2_ released into the atmosphere is increasing annually (Abrantes et al., 2021). On the other hand, replacing fossil fuels with green energy resources could not be carried out in the near future. Thus, stopping of CO_2_ emissions into the atmosphere could not be an operational approach, at least in the present decade (York and Bell, 2019). Instead, researchers have found different ways to store or use such polluting species (Yang et al., 2021; Liu et al., 2022; Pandey et al., 2022). Artificial photosynthesis (Keijer et al., 2021), electrochemical reduction (Lin et al., 2019; Zhao and Quan, 2021), and also chemical reactions (Pramudita and Motokura, 2021; Porgar and Rahmanian, 2022) are some of those approaches.

At least at the present time, it seems that chemical reactions might be one of the most practical approaches for reduction of CO_2_ (Sharghi et al., 2017; Santos-Carballal et al., 2021). The chemical reactions could be run in the absence of electrical energy or fragile systems, which are required for artificial photosynthesis.

Previous reports reveal that some of the borane derivatives (in the presence of catalysts) could be a choice for chemical reduction of CO_2_ due to their ability to release negative hydrogen atoms. As an example, in recent years, Kadota et al. (2019) have successfully used metallic borohydrides in the presence of triphenylphosphine as the catalyst and acetonitrile as the solvent to give a porous coordination polymer (Kadota et al., 2019). In another work (2020), they used calcium borohydrides in the presence of pyridine derivatives as the catalyst and dimethylsulfoxide (DMSO) as a solvent at 40°C to consume CO_2_ for the production of calcium formate (Kadota et al., 2020). In addition, Zhu et al. (2019), reacted lithium and sodium borohydrides to CO_2_ in a gas–solid phase system for 24 h to yield hydrogen molecule and trimethylborane (Zhu et al., 2019). Also, there are some other examples in which metallic borohydrides, mostly in the presence of the base and solvent, have reacted to CO_2_ to yield synthesized composites or other products (Fujiwara et al., 2014; Bontemps, 2016). In addition, sensing or attracting CO_2_ by special nano-structures *via* chemical reactions was studied and proved to be performable in some previous reports. It indicates that some of the electron-rich components are able to reduce this gaseous species (Siadati et al., 2016a; Pakravan and Siadati, 2017; Vessally et al., 2017; Kadhim et al., 2022a; Kadhim et al., 2022b; Li and Zhao, 2022). Also, the findings of the previous reports show that studying the mechanism of the reactions by using the PES method and reaction pathways approach could give valuable information about the fate of the atomic and molecular interactions between the chemical species (Siadati, 2015; Siadati et al., 2016b; Mohtat et al., 2018; Kula et al., 2021; Vessally and Hosseinian, 2021).

Due to the abovementioned issues, in this work, we attempted to follow each of the possible reaction subways between CO_2_ and borane in order to find the fate of this system. The results of the PES studies and also the molecular dynamic simulations showed that formic acid (and further metaboric acid production from transformation of boric acid) would form finally from the reaction between CO_2_ and borane, at least in the gas phase.

## Calculation details

After performing the molecular dynamic simulations for the atomic system (containing boron, carbon, 3*hydrogen, and 2*oxygen), a number of meta-stable species (which might emerge *via* different atomic orientations) were detected. Then, after design and optimization of each separated molecule (BH_3_, and CO_2_), any interaction that could occur (despite energy peaks and any other barrier) was considered and input into the software to give any possible reaction pathway. After extracting the results *via* optimization of each atomic state, various reaction pathways with separated or common meta-stable species were designed. Those species consisted of reactant complexes, transition states (TSs), intermediates, and final products. The Gaussian 03 chemical quantum package (Frisch et al., 2003) was applied to perform the calculations, and the density functional theory (DFT) procedure at the B3LYP/6-311++G (d,p) level of theory was used to optimize the possible structures (Becke, 1988; Lee et al., 1988). The TS structures were recognized by using the synchronous transit-guided quasi-Newton (STQN) approach (Peng et al., 1996). In addition, in order to find the electrical charge of each atom in the reactants, intermediates, products, and TSs, the natural bond orbital (NBO) analysis was used (Reed et al., 1988).

The following formula was applied for calculating the global electron density transfer (*GEDT*):

$$GEDT=\sum q_{A}.$$ (1)

In which q_A_ is the net atom in molecule (AIM) charge (calculated by AIMAll (Version 10.07.01) (Keith, 2010; Domingo et al., 2021)) and the sum covered the entire atoms of CO_2_ species.

Also, molecular dynamic simulations were performed to investigate the behavior of the mentioned atoms (Lawan et al., 2022).

## Results and discussion

**FIGURE 1:**
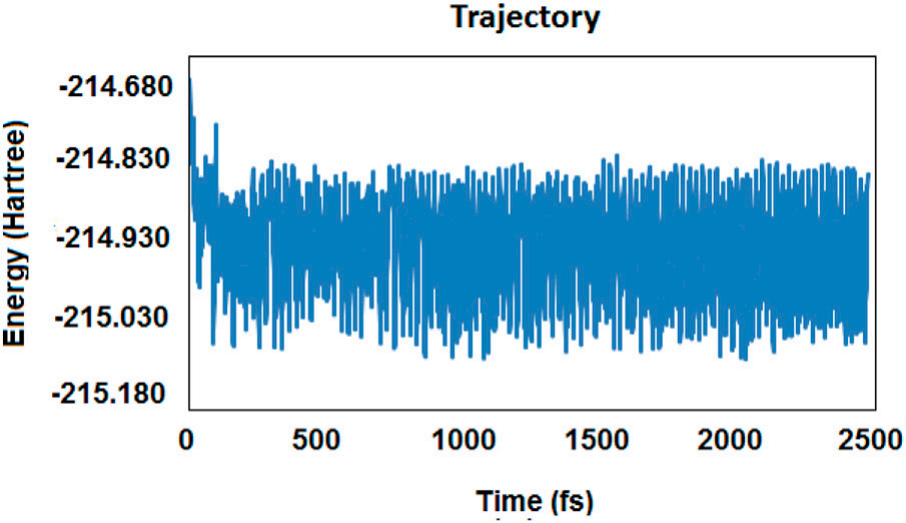
Results of the trajectory diagram for the molecular dynamic simulation based on energy per time (in femtosecond) (fs).

To investigate the possibility of CO_2_ reduction by the gaseous form of borane (which evaporates at about 65°C to 67°C), all possible interactions between those two species were designed in order to make an accurate map. There were several reaction pathways with different orientations, intermediates, and products as the final state of energy. Moreover, a number of species came from different routes, which could form the cycles. The results of the molecular dynamic simulations helped us recognize some of the species that might emerge during the reaction coordinate (Figure 1). The simulation part of the calculations was performed by using Gaussian 03 software. The classical trajectory calculation using the Born–Oppenheimer molecular dynamics model (BOMD) was performed in about 2473 femtoseconds (fs) (as the total simulation time). The force field was calculated with the aid of the DFT method; the temperature was set at 400 K, and the time-step was about 0.618 fs.

**FIGURE 2:**
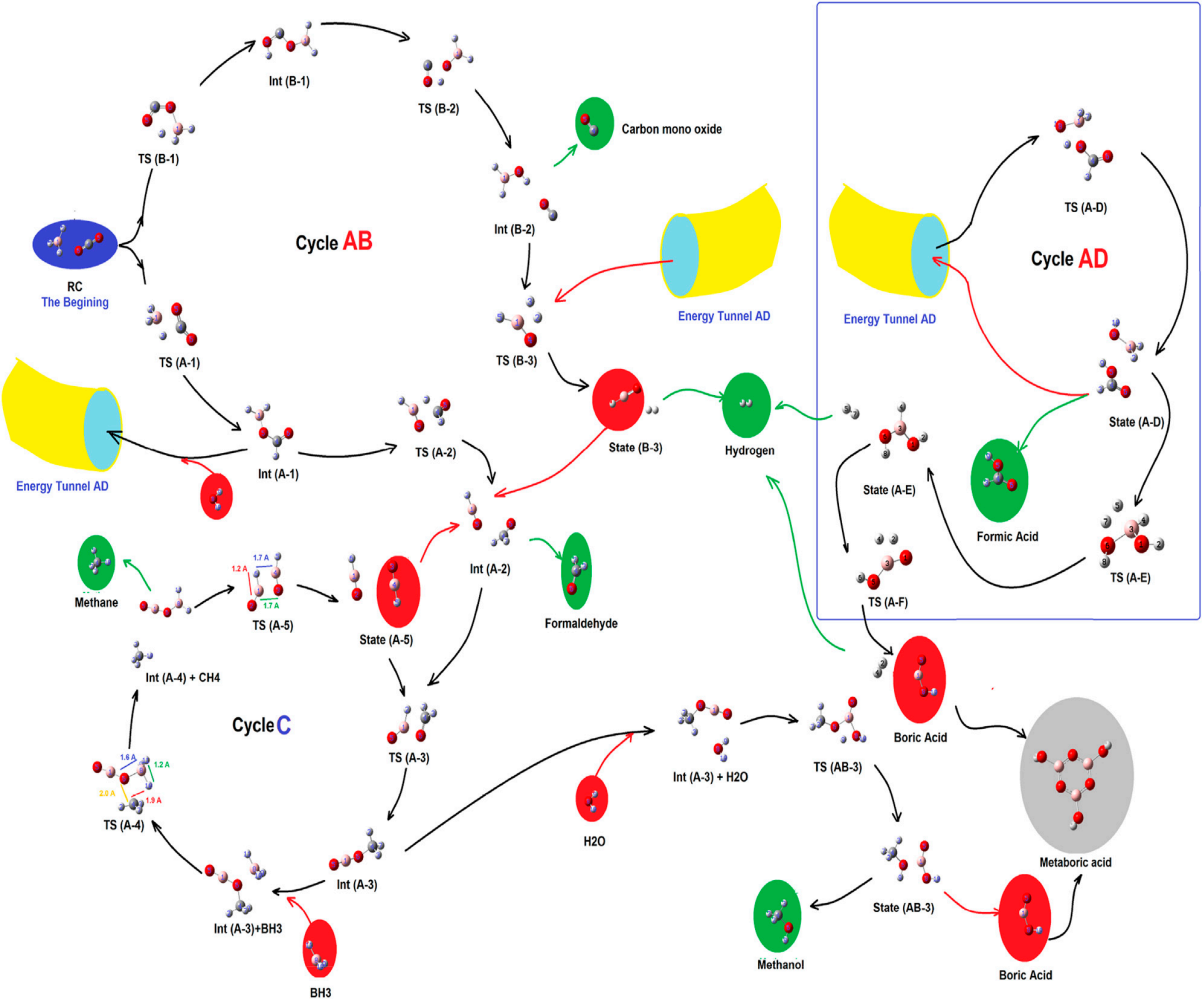
Complex of the main routes and sub-branched reaction valleys for the reduction process of CO_2_ by borane in gas phase.

**SCHEME 1:**
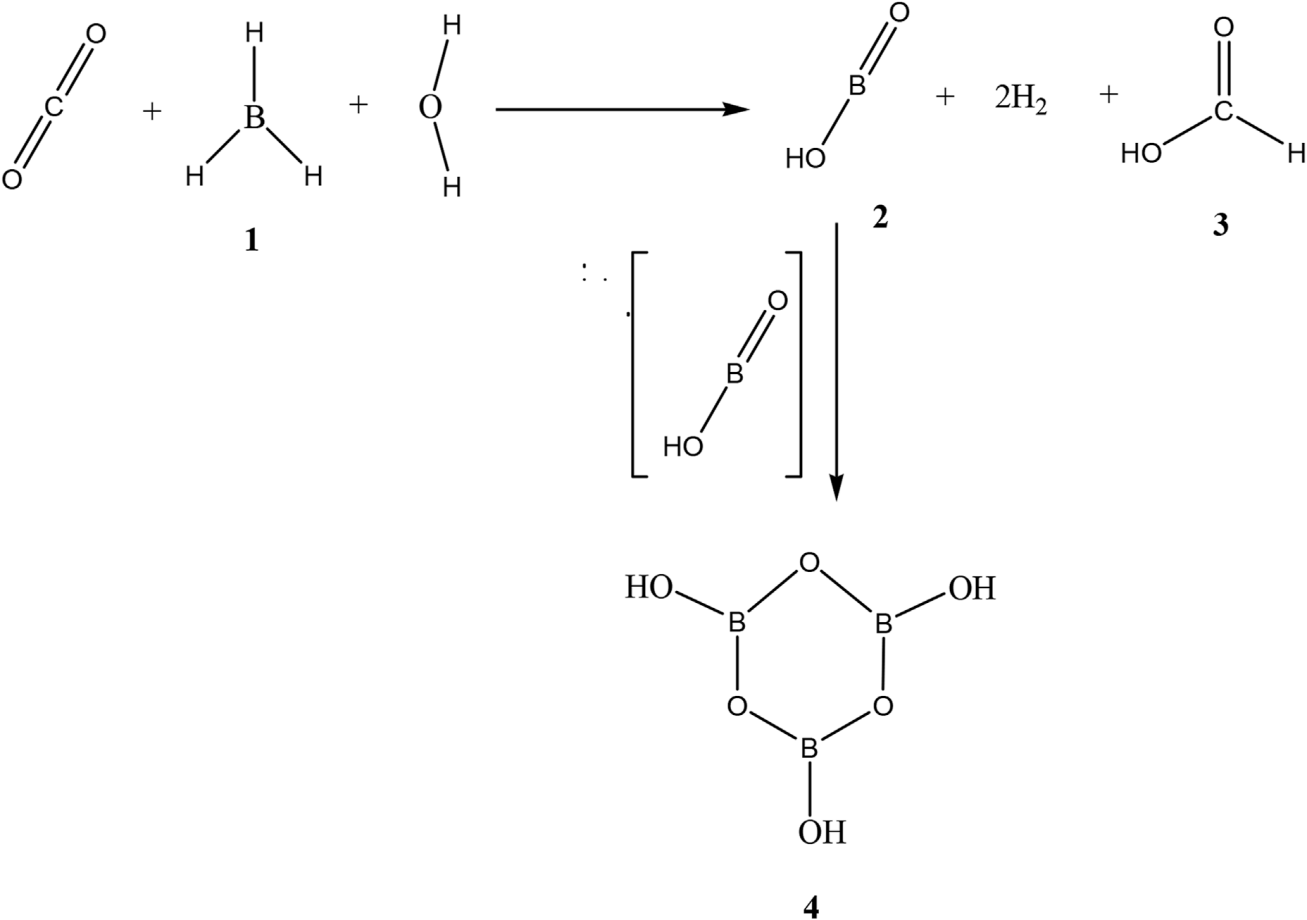
Total scheme of the reaction between CO_2_ and borane **1,** leading to production of formic acid **3,** mono boric acid **2**, and metaboric acid trimer **4**.

As shown in Figure 2 (Scheme 1), there were two primitive routes which produced a number of different reaction valleys (as sub-branches). Each of those sub-branches reaching a product or was linked to a cycle.

As shown in Figure 2, there are three main cycles which are linked to each other, by pillars of energy. Those are cycles AB, AD, and cycle C, which produce carbon monoxide, molecular hydrogen, and formaldehyde (cycle AB); methane and methanol (cycle C); and formic acid (cycle AD), respectively. At the beginning, there were no compounds except CO_2_, and BH_3_, while after the production of two primitive routes (containing routes A and B), and the formation of cycle AB, several meta-stable species with a variety of energy barriers, peaks, and valleys have emerged. Each of those would be described in the following sub-sections.

### Cycle AB, reaction channels, and products

**FIGURE 3:**
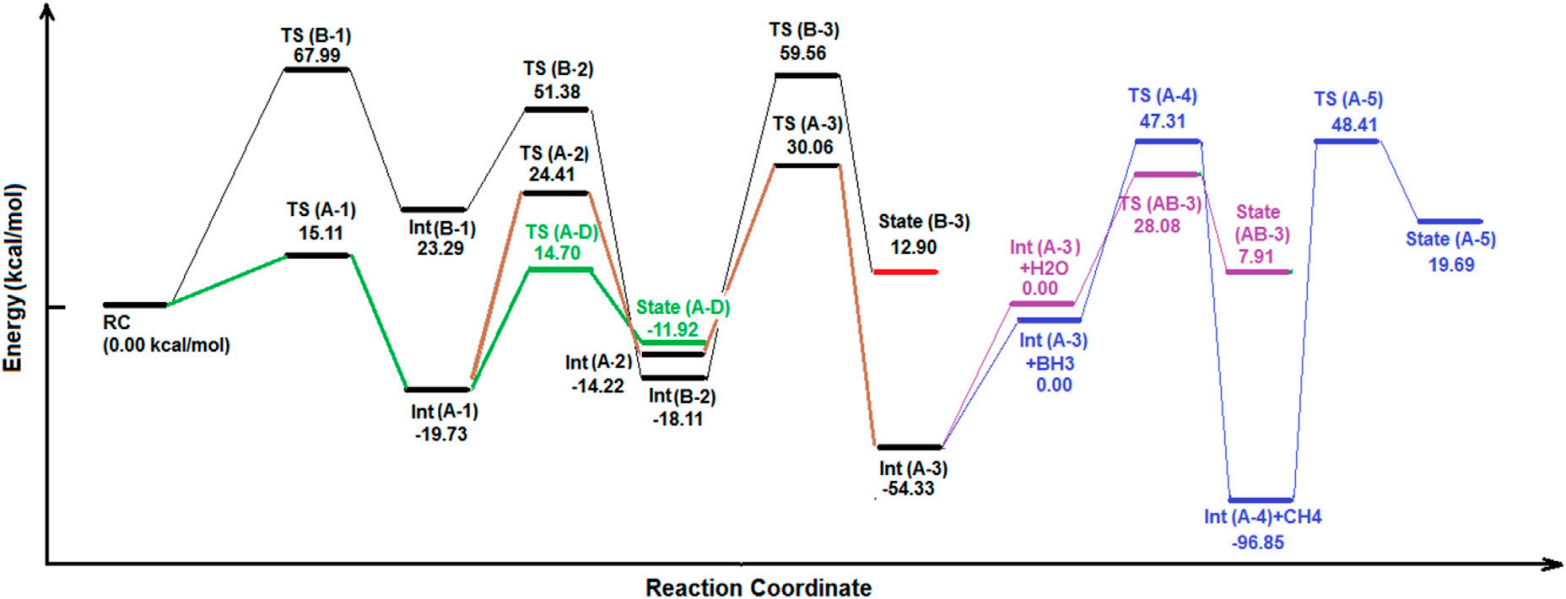
Energy diagram based on the reaction progress at B3LYP/6-311++G (d,p) level of theory.

As shown in Figures 2, 3, at the first step, the primary reactant complex (containing CO_2_, and BH_3_) approaches each other in different orientations, which causes two separated main routes to emerge (channels A and B as well as the formation of cycle AB). Channel A is produced when 1 C=O bond of CO_2_ comes closer to one B–H bond of borane (a side-by-side position) to produce **TS (A-1),** with an energy barrier of 15.11 kcal mol ^−1^ (making this process possible in view of energy). Also, channel B emerges *via* formation of a five-membered ring (**TS (B-1**)) during proton transfer from the boron atom of BH_3_ to the free oxygen of CO_2_. The energy barrier of this species is about 67.99 kcal mol ^−1^, which shows that route B needs a lot of thermal energy; somehow, in usual temperatures and in ambient systems, hypothetical channel A is formed much faster than its parallel route (channel B). Despite the fact that channel B is nearly banned at the energy barrier of **TS (B-1)**, it is mentioned that the subsequent stages of that route contain **Int (B-1)** (23.29 kcal mol ^−1^), **TS (B-2)** (51.38 kcal mol ^−1^; barrier = 28.09 kcal mol ^−1^), **Int (B-2)** (-18.11 kcal mol ^−1^), **TS (B-3) (**59.56 kcal mol ^−1^; barrier = 77.67 kcal mol ^−1^), and **state (B-3)** [releasing a molecular hydrogen and a HBO species into cycle C *via* **Int (A-2)**]. Thus, channel B is forbidden from the kinetic point of view. **Int (B-2)** releases a carbon monoxide molecule before the formation of **TS (B-3)**. Also, the results of the calculations show that in **TS (A-1)**, the amount of GEDT is about −0.242 (indicating the global electron transfer from CO_2_ to borane, while the amount of this parameter for **Int (A-1)** is about −0.847 (attacking the electron density from borane segment to CO_2_).

In channel A, **TS (A-1)** (15.11 kcal mol ^−1^) transforms to **Int (A-1)** (−19.73 kcal mol ^−1^), **TS (A-2)** (24.41 kcal mol ^−1^; barrier = 44.14 kcal mol ^−1^), and **Int (A-2) (**−14.22 kcal mol ^−1^), which completes cycle AB. Also, it seems that reaching **Int (A-2)** *via* channel A is difficult, but this route is still favored compared to channel B. Subsequently, **Int (A-2)** could release formaldehyde or transform **TS (A-3)** to form cycle C. It must be noted that at the middle of channel A, **Int (A-1)** could react with a single water molecule and pass through the reaction pathway AD and form **TS (A-D)**, having a relative energy barrier of 14.70 kcal mol ^−1^. Thus, moving **Int (A-1)** and H_2_O to the reaction pathway A–D could give valuable information about the fate of the reaction cycles of the CO_2_–borane complex.

### Cycle C, reaction channels, and products

Cycle C would begin with the transformation of **Int (A-2)** into **TS (A-3)**. In this case, the formaldehyde part of **Int (A-2)** moves to reach a parallel situation with HBO species; somehow, the H–B bond of HBO approaches the O=C bond of formaldehyde. Then, the proton of HBO is transferred to the carbon of formaldehyde to yield **Int (A-3),** which is extremely stable even in comparison with RC (-54.33 kcal mol ^−1^). For the next step of this cycle, another BH_3_ species attacks **Int (A-3)** and forms an **Int (A-3)-BH3** system. Then, it receives energy and turns into **TS (A-4)** with an energy content of 47.31 kcal mol^−1^ compared to the **Int (A-3)-BH**_**3**_ system (which could only proceed in high temperatures or some irradiative environments). In addition, this TS loses energy and reaches the **Int (A-4) + CH**_**4**_ system with a relative PES of -96.85 kcal mol^−1^ (the lowest energy of whole cycles). It shows that in spite of high activation energies, which makes it kinetically unfavorable (at least in ambient conditions), the formation of some products of these cycles is extremely favored in view of thermodynamics. Then, the **Int (A-4)+CH**_**4**_ complex releases a methane molecule, and Int (A-4) (H_2_BOBO) decomposes into two equal HBO fragments (**state (A-5**), with an energy content of 19.69 kcal mol^−1^ compared to **Int (A-4))** *via* the **TS (A-5)** [48.41 kcal mol^−1^ compared to **Int (A-4)**]**.** Finally, both HBO fragments of **state (A-5)** return to the **Int (A-2)** system to supply and re-start the cycle C. Thus, by circulation of cycle C, a BH_3_ is consumed and a methane molecule is released. However, most peaks of cycle C are extremely energy-demanding and require much energy.

### Cycle AD, reaction pathways, and products

The results of the molecular dynamic simulations and the PES studies by means of DFT calculations indicate that the most important cycle of the whole system is cycle A–D. As shown in Figures 2, 3, **Int (A-1)** could receive a water molecule and form an **Int (A-1)**-H_2_O) complex. Then, this complex gains energy and forms TS (A-D) *via* the reaction pathway AD, with a relative PES of 14.70 kcal mol^−1^ [compared to the **Int (A-1)**-H_2_O complex]. This process is being carried out *via* an electron pair attack from the oxygen of water molecules to the boron atom of **Int (A-1)**. The process continues by proton transfer from the H_2_O^+^ fragment to the formate part. By following these changes, **state (A-D)** forms *via* quenching **TS (A-D)**. Two species contain a formic acid molecule and a H_2_BO fragment in **state (A-D)** (−11.92 kcal mol^−1^). After completion of cycle AD, the H_2_BO meta-stable species is released from that cycle *via* reaction path AD to supply **TS (B-3)** of cycle AB. Moreover, formic acid is released, which is both kinetically and thermodynamically favorable. It shows that among all reaction valleys and cyclic complexes, the route in which **Int (A-1)** from cycle AB and forms cycle AD to release formic acid is the most favorable one. Also, there are several reports revealing the fast formation of the six-membered metaboric acid from the transformation of boric acid (Grigorovskaya et al., 2009; Balcı et al., 2012; Hoffendahl et al., 2014).

**FIGURE 4:**
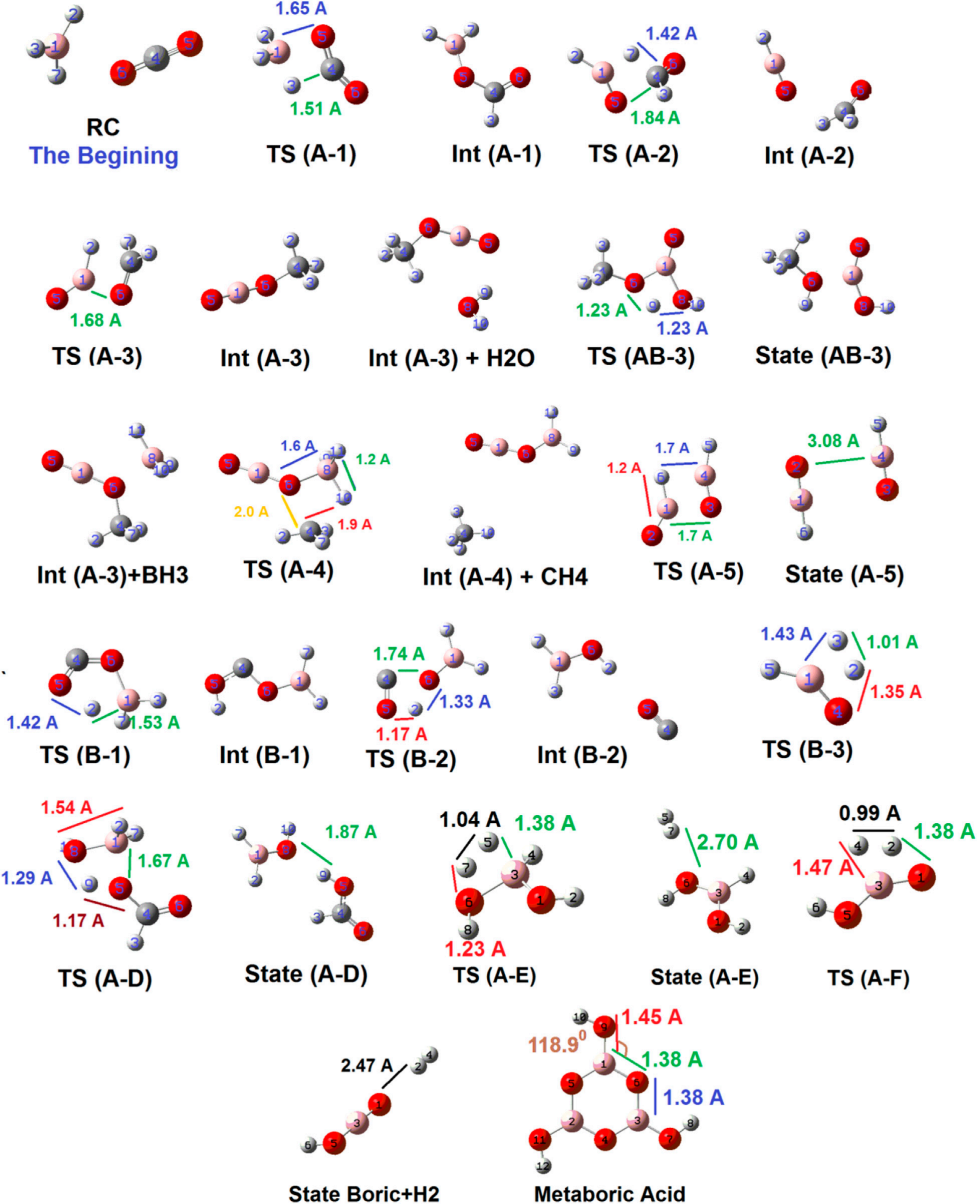
Geometrical structures of all detected species which emerge during the reaction coordinate optimized at B3LYP/6-311++G (d,p) level of theory.

Figure 4 shows the geometrical structures of several detected species which might emerge during the reduction reaction of CO_2_ by BH_3_ species. As shown below, the two reactants approach each other in a unique orientation to form the reactant complex (RC). Then, RC receives energy up to about 15.11 kcal mol^−1^ to form **TS (A-1),** which is significantly favorable compared to **TS (B-1)** (67.99 kcal mol^−1^). During this transformation, B (1)---H (3) bond is breaking [from 1.85 Ǻ in RC to 1.3 Ǻ in **TS (A-1)**], while B (1)---O (5) (1.65 Ǻ) as well as C (4)---H (3) (1.51 Ǻ) bonds are forming. Then, TS (A-1) loses energy (34.4 kcal mol^−1^) to form **Int (A-1)**. This species gains energy (44.14 kcal mol^−1^) to form **TS (A-2)** in which the C (4)---O (5) bond length reaches to 1.84 Ǻ to break totally (as well as transferring H (7) from B (1) to C (4). Following it, **Int (A-2)** forms, which could release an aldehyde or begin a cycle (C). Alternatively, **Int (A-1)** could react with a water molecule and gain energy to reach **TS (A-D)** *via* the reaction pathway (A-D). As shown in Figure 4, O 8) of the water molecule approaches B (1) and forms **TS (A-D)**. In this species, the distance of the O (8)---B 1) forming bond is about 1.54 Ǻ, while the distances of the B (1)---O (5), and the O (8)---H 9) breaking bonds are 1.67 Ǻ, and 1.29 Ǻ, respectively. It shows that the bond distances in the transition state are more likely to product than to intermediate. The PES data given in Figure 3 confirm these results. Also, it should be noted that **TS (AB-3)** could potentially be considered for the (York and Bell, 2019; Herndon and Whiteside, 2021)-proton-sigmatropic shift reaction due to properties which have recently been revealed in Jasiński, (2020). We have used the B3LYP/6311++G (d,p) level of theory for these mechanism studies due to the fact that this basis set has been widely applied for theoretical prediction of the experimentally investigated reaction parameters. The results of our theoretical studies on the reaction parameters had very good agreement with the previous experimental reports (Siadati et al., 2011; Bekhradnia et al., 2014; Siadati, 2016).

### Total circulation of the complex of cycles

The results of the relative PES and the molecular dynamic simulations indicate that the least energy pathway for the reaction between borane and CO_2_ (the green line in Figure 3) is the reactant complex (**RC**) to **TS (A-1)** to **Int (A-1)** to **TS (A-D)** to formic acid with a relative PES of 0.00 kcal mol^−1^, +15.11 kcal mol^−1^, -19.37 kcal mol^−1^, and +14.70 kcal mol^−1^, respectively. Thus, among all possible species, formic acid emerges finally from the reaction between CO_2_ and borane, at least in the gas phase.

As shown in Table 1, the negative frequencies of TSs (A-1) and (A-D), are -457.90, and -1262.52 cm^−1^, respectively. These strong negative frequencies confirm the reliability of the calculations of the TSs. Also, the ΔG^#^ (Gibbs free energy difference) and the ΔH^#^ (enthalpy difference) for the formation of TS (A-1) and TS (A–D) are 14.79 kcal mol^−1^, 19.54 kcal mol^−1^, 17.57 kcal mol^−1^, and 18.94 kcal mol^−1^, respectively. These values indicate the relatively low energy barriers and favorability of the formation of the mentioned TSs in view of thermodynamics, leading to the production of formic acid on the one hand, and metaboric acid, on the other hand.

**TABLE 1:** Thermodynamic parameters as well as the negative frequency values of TSs for the possible reaction route.

TS	ΔG^#^ (kcal mol^−1^)	ΔH^#^ (kcal mol^−1^)	ΔS^#^ (cal mol^−1^ K^−1^)	Negative freq (cm^−1^)
TS (A-1)	14.79	19.54	−15.93	−457.90
TS (A-D)	17.57	18.94	−4.57	−1262.52

## Conclusion

In the present research, we attempted to follow each of the possible pathways for all of the interactions between CO_2_ and borane in order to find the fate of the reaction. The results of the PES studies and also those of the molecular dynamic simulations show that the behaviors of borane and CO_2_ lead to the emergence of several possible stable and meta-stable species. Some molecular fragments which are linked to each other by energetic routes and make some related cycles.

The result also indicate that the least energy pathway for the reaction between borane and CO_2_ is **RC** to **TS (A-1)** to **Int (A-1)** to **TS (A-D)** to formic acid with relative PESs of 0.00 kcal mol^−1^, +15.11 kcal mol^−1^, −19.37 kcal mol^−1^, +14.70 kcal mol^−1^, and -11.92 kcal mol^−1^, respectively. Thus, between all possible species which emerge from the abovementioned complex of cycles, formic acid (and further metaboric acid production from the transformation of boric acid) is formed eventually, at least in the gaseous atmosphere.

Finally, due to the energy barriers and valleys, reduction of CO_2_ by borane molecules is possible and even favorable, at least in gas phase. This suggests that the use of gaseous borane (boiling point = 67°C) for reduction of CO_2_ should be taken under further consideration by scientists. Investigating any probable meta-stable species (which might emerge during the designation of the whole complex of the reaction cycles) would be a reliable way for understanding the behavior of molecules in the gas phase. Thus, the outcome of such a project could be helpful for further research in controlling the amount of atmospheric CO_2_.

## Data Availability

The original contributions presented in the study are included in the article/Supplementary Material; further inquiries can be directed to the corresponding author.
